# Thiamin addition to soil increases potato tuber thiamin content under greenhouse conditions

**DOI:** 10.7717/peerj.20684

**Published:** 2026-01-29

**Authors:** Aymeric Goyer, Ravi Phillips, Aidan Seidel, David Handy, Jeffrey C. Anderson, Andrea Schiffer, Alexandra J. Weisberg, Paul C. Bethke

**Affiliations:** 1 Botany and Plant Pathology, Oregon State University, Corvallis, OR, United States of America; 2 United States Department of Agriculture—Agricultural Research Service, Madison, WI, United States of America

**Keywords:** Thiamin, Potato, Soil, Yield, Biofortification

## Abstract

Thiamin is essential for human health, but humans do not synthesize it and must consume it through regular dietary intake. Plants synthesize thiamin in photosynthetic tissues to support various primary metabolic pathways. In addition, plants can also absorb thiamin from the soil. Interestingly, plant growth can be improved by supplying exogenous thiamin, but this effect has not been investigated in potato (*Solanum tuberosum*). Here, we report the effect of soil thiamin amendment on yield and tissue thiamin content of three potato varieties grown in a greenhouse. We watered plants with different concentrations of thiamin on a weekly basis from emergence until harvest. Under optimal growth conditions, thiamin supplementation did not affect tuber yield, regardless of soil type. Thiamin accumulated up to 58, six and three times in roots, tubers and stems, respectively, of plants grown in soil supplemented with thiamin compared to control plants, while leaf thiamin content did not significantly change. Quantitative polymerase chain reaction analysis showed that soil thiamin supplementation had no or little effect on the expression of two key thiamin biosynthesis genes in leaves. Our results indicate that increasing soil thiamin concentration does not improve potato yield under optimal growth conditions at the greenhouse scale. However, increased yield at field-scale under stress conditions remains to be tested. Intriguingly, the accumulation of thiamin in potato tubers suggests that soil thiamin supplementation may be a viable strategy for biofortification.

## Introduction

Thiamin is a water-soluble vitamin (B_1_). In its pyrophosphorylated form, it serves as an enzymatic cofactor in universal metabolic pathways such as glycolysis, the tricarboxylic acid cycle, and the pentose phosphate pathway. Plants produce thiamin *de novo* in photosynthetic tissues (Goyer, 2010). In addition, plants can also absorb thiamin from the soil and transport it to above-ground tissues (Mozafar & Oertli, 1992). This dual ability to both synthesize and obtain thiamin from the environment may provide a selective advantage by cutting energy costs when thiamin is readily available in the environment. Indeed, thiamin biosynthesis is energetically expensive because the first two enzymes of the pathway, THI1 (also referred to as THI4 in the literature) and THIC, are highly inefficient, losing their catalytic activities after one and about five catalytic cycles, respectively (Chatterjee et al., 2011; Hanson et al., 2018; Palmer & Downs, 2013). Because of these inefficiencies in thiamin biosynthesis, the uptake of thiamin from soil may contribute to the total thiamin pool in plant tissues and reduce the need for *de novo* thiamin biosynthesis, thereby enabling plants to save energy that they can use for biomass production. Along these lines, simulations of crop growth estimated that eliminating the costs associated with rapid turnover of THI1 and THIC could increase biomass yield by as much as 4.2% in annual crops (Hanson et al., 2018).

Despite predictions that thiamin supplementation may benefit plant health, only a handful of studies have directly tested its effects on crop yield. Spraying foliar tissues with thiamin has been reported to increase potato yield (Iijima, 1960), yet application of thiamin in this manner has not been widely adopted to date, most likely due to high costs of producing synthetic thiamin. An alternative and likely more cost-effective approach is to amend soils with thiamin through natural sources. For instance, soils can be amended with sewage sludge and sewage water that contain high concentrations of thiamin (Lemmer & Nitschke, 1994; Mozafar, 1994; Srinath & Pillai, 1966; Von Kocher & Corti, 1952), and green manures are another potential rich source of thiamin. In addition to these fertilizers, soil microorganisms are another source of thiamin. Indeed, many soil bacteria and fungi synthesize thiamin *de novo* and release it into the environment (Strzelczyk & Rozycki, 1985). Several studies have shown that exogenous application of thiamin to the rhizosphere increased yield, although this effect varied between plant species (Bonner & Greene, 1938; Bonner & Greene, 1939; Sivadjian, 1953). To our knowledge, no published studies have yet addressed the yield response of potatoes to soil-applied thiamin.

The uptake and transport of exogenously supplied thiamin might also lead to thiamin-enriched edible parts of plants, which would be of interest for crop biofortification in the fight against hidden malnutrition (Goyer, 2017). However, the fate of thiamin taken up by plants from the growth media has seldom been investigated. Experiments in seedlings of soybean have shown that thiamin taken up by roots from the growth media is transported throughout the plant, but the distribution varies depending on supplied thiamin concentrations (Mozafar & Oertli, 1992; Mozafar & Oertli, 1993). There is currently no available data for biofortification of thiamin in potatoes.

The goal of this study was to determine whether soil amendment with thiamin increases potato yield and produces thiamin-enriched potatoes. As a proof-of-concept, we applied synthetic thiamin to soil under greenhouse conditions. This study provides new information on thiamin uptake from soil and transport in potato and its effect on yield, and suggests that thiamin soil amendment is a viable strategy for potato biofortification.

## Materials & Methods

### Chemicals

Thiamin hydrochloride was purchased from Millipore Sigma (Burlington, MA, USA).

### Plant growth

Plants were grown in a greenhouse with temperatures set at 21 °C day/18 °C night. High-pressure sodium lamps provided supplemental light to maintain a minimum of 14 h photoperiod. The greenhouse was equipped with four 6-m long × 1.4-m wide benches. Beneficial organisms (*Amblyseius cucumeris*, *Orius*, and *Swirskii* predatory mites) were released once a week to control for thrips. In spring/summer 2023, there was a thrips outbreak that required applying insecticide (Xxpire; Corteva, Johnston, IA, USA).

For the spring/summer growing season 2023 (Table 1), seed tubers of the potato variety Clearwater Russet (medium to late maturing) were obtained from the Oregon State University Klamath Basin Research and Extension Center. Tubers were cut into 28–40 g seed pieces and suberized for five days before planting approximately 10 cm deep in 11.3-liter pots (Elite Nursery Containers 1200 Series Black; Greenhouse Megastore, Danville, IL, USA) containing a mixture of Sunshine Mix #4 (Sun Gro Horticulture, Agawam, MA, USA), sand, and slow-release fertilizer (Osmocote Plus; The Scotts Miracle-Gro Company, Marysville, OH, USA). Pasteurized soil was prepared by mixing one bag of Sunshine Mix #4 (79 l) and one bag of sand (14 l) in a concrete mixer, adding water until moist, and pasteurizing the mixture with two cycles of 30 min at 180 °C. After cooling, the pasteurized soil mixture (102 l) and 537 cm^3^ of Osmocote Plus (The Scotts Miracle-Gro Company) were homogenized in a concrete mixer. Pots were filled to within 2.5 cm of the top with the prepared soil mix. For non-pasteurized soil, we mixed Sunshine Mix #4 (Sun Gro Horticulture), sand, and Osmocote Plus (The Scotts Miracle-Gro Company) directly in the concrete mixer in the same proportion as pasteurized mixture. The average weight ± SD of the pots filled with soil was 7.7 ± 0.36 kg. There was a total of 50 pots arranged in two rows of 17 pots and one row of 16 pots on one bench. Pots were ∼30-cm apart within rows (center to center), and ∼91-cm apart between rows. Plants were hand-watered as needed, typically every 2–3 days for the first few weeks after emergence, then every day.

**Table 1:** Dates of planting, emergence, first and last thiamin application, and harvest.

**Variety**	**Soil type**	**Planting date**	**Emergence**	**First and last thiamin application**	**Harvest date**
***Spring/summer 2023***					
Clearwater Russet	Sunshine Mix#4, sand (4:1)	24 April	6 May (<10%) 15 May (100%)	22 May/16 August	24 August
***Winter/spring 2024***					
Russet Norkotah	Sunshine Mix#4	29 January	14 February (15%) 19 February (68%) 29 February (100%)	29 February/18 April	26 April
***Spring/summer 2024***					
Snowden	Adkins	10 April	22 April (22%) 25 April (83%) 1 May (96%)	8 May/1 August	7 August
Clearwater Russet	Adkins	19 April	1 May (46%) 16 May (98%)	16 May/15 August	19 August

For the winter/spring growing season 2024 (Table 1), seed tubers of the variety Russet Norkotah were obtained from plants grown during spring/summer 2023 under the conditions described above. Seed tubers were cut and suberized as described above and planted as described above with the exception that sand was not included in the soil mixture. There was a total of 76 pots arranged in four rows of 19 pots on one bench. Pots were ∼30-cm apart within and between rows (center to center). Plants were hand-watered as needed as described above.

For the spring/summer growing season 2024 (Table 1), Clearwater Russet seed tubers were obtained from the Oregon State University Klamath Basin Research and Extension Center. Seeds of the variety Snowden were received from Eagle River Seed Farm (Eagle River, WI, USA). Seed tubers were harvested in early October 2023 and stored at 3.3 °C and 95% relative humidity. Before planting, tubers were cut and suberized as described above. Seed tubers were planted in Adkins soils. Soil was collected from the top 30-cm layer of a non-cultivated, Rye grass field from Umatilla County, Oregon (45.817318°N–119.294922°W). Soil was mixed with Osmocote Plus (The Scotts Miracle-Gro Company) in a concrete mixer at the same ratio as described above and transferred to 11.3-liters pots (∼36 kg per pot). For each variety, there were 54 pots arranged in three rows of 18 pots each per bench (one variety per bench). Pots were ∼30-cm apart within rows (center to center), and ∼91-cm apart between rows. Soil moisture content was monitored with Terros 12 sensors connected to a cloud data logger ZL6 (METER, Pullman, WA, USA). There were two to three sensors per bench. Plants were hand-irrigated to try to maintain a 65–80% soil water holding capacity from planting to pre-emergence, 70–80% soil water holding capacity from emergence to tuber initiation, 80–90% soil water holding capacity from tuber initiation to plant senescence, 60–65% soil water holding capacity from plant senescence to harvest. Data on soil temperature, % water holding capacity, and electroconductivity are in Table S1.

### Thiamin soil supplementation

Soil was supplemented with thiamin on a weekly basis starting shortly after full emergence until close to harvest (see Table 1 for first and last treatment dates). There were up to five thiamin supplementation rates: (1) no thiamin supplementation (0X); (2) 39 mg thiamin per pot (1X); (3) 195 mg thiamin per pot (5X); (4) 780 mg thiamin per pot (20X); (5) 1,950 mg thiamin per pot (50X). Thiamin was dissolved in water and added to each pot in 1-liter volume for the trials done in spring/summer 2023 and winter/spring 2024, and in 100-ml volume for the trial done in spring/summer 2024. There was no or little drip from the pots after thiamin treatment. Plants were watered the rest of the week as needed avoiding any excessive drip from the pots.

For the spring/summer growing season 2023, there were five treatments: “no pasteurization—no thiamin”, “pasteurization —no thiamin”, “pasteurization—1X thiamin”, “pasteurization—5X thiamin”, “pasteurization –20X thiamin”. Each treatment was replicated ten times (pots are experimental units) with the exception of treatments “no pasteurization—no thiamin” and “pasteurization—20X thiamin” for which there were 11 and nine replicated pots, respectively. Pots were arranged in a completely randomized design (Fig. S1).

For the winter/spring growing season 2024, there were six treatments: “no pasteurization—no thiamin”, “no pasteurization—20X thiamin”, “no pasteurization—50X thiamin”, “pasteurization—no thiamin”, “pasteurization—20X thiamin”, “pasteurization—50X thiamin”. Each treatment was replicated four times (groups of three plants are experimental units) in a randomized complete block design (Fig. S1). In addition, treatments “pasteurization—no thiamin” and “pasteurization—20X thiamin” had one extra pot each randomly distributed, and treatment “pasteurization—50X thiamin” had two extra pots randomly distributed.

For the spring/summer growing season 2024, there were three treatments: “no pasteurization—no thiamin”, “no pasteurization—20X thiamin”, and “no pasteurization—50X thiamin”. Each treatment was replicated six times (groups of three plants are experimental units) in a randomized complete block design (Fig. S1).

### Thiamin extraction and quantification

#### Thiamin extraction from tubers

Immediately after harvest, we selected two tubers per plant (spring/summer 2023 and winter/spring 2024) or randomly selected two tubers per group of plants from three random groups per treatment (spring/summer 2024) that we washed with water and let dry at room temperature. Then, we cut each tuber in 1/16th wedges that we chopped, immediately froze in liquid nitrogen, lyophilized and ground to powder. Thiamin was extracted from 100 mg of tuber powder in 4 ml 0.1 N HCl in 10-ml glass tubes. Samples were homogenized by vortexing and placed in a sonicator for 10 min. A 1-ml aliquot was then transferred to a 1.7-ml Eppendorf tube and centrifuged at 14,000×*g* for 10 min to pellet starch. The supernatant was transferred to a centrifugal filter device (Nanosep 3K Omega; Pall Corporation) and centrifuged for 30 min at 14,000×*g*. This latter step was eventually omitted, which had no effect on the chromatography and quantification. The flow-through was transferred to a new tube and used for thiamin oxidation into thiochrome by adding 17 µl of oxidizing solution (10 mM K_3_Fe(CN)_6_ prepared in 3.7 N NaOH) and 34 µl of methanol to 100 µl of extract. Oxidized extracts were centrifuged for 10 min at 14,000×*g* and the supernatant was transferred to amber HPLC vials.

#### Thiamin extraction from leaves, roots, and stems

Thiamin was extracted from 15 mg of freeze-dried material as described above. Stems with attached leaves (four per treatment) from the top third of the plants were collected 24 h after thiamin treatment from the varieties Snowden and Clearwater Russet on 18 July, 2024. Leaflets (two per leaf) were removed from leaves attached to the collected stems. For stems, 2–3-cm long sections were cut off with a blade. Roots were collected at harvest, thoroughly washed with water and patted dry. All tissues were placed in 15- or 50-ml conical tubes that were immediately frozen in liquid nitrogen and stored in a −80 °C freezer until used for lyophilization and analysis.

#### Thiamin quantification

The thiochromes of thiamin, thiamin monophosphate and thiamin diphosphate were separated by HPLC and quantified using a standard curve as described before (Dong, Stockwell & Goyer, 2015). Recoveries were determined by spiking samples with an equal amount of thiamin and calculated as follows: R (%) = ((amount in sample + spike)—(amount in sample))/amount in spike * 100. Recoveries were 82% for roots, 52% for leaves, and 61% for tubers. Data presented below are not corrected for recovery. The variability in recovery between tissues does not affect quantitative comparisons between soil thiamin treatments within each tissue type.

### Quantitative reverse transcriptase polymerase chain reaction

The Minimum Information for Publication of Quantitative Real-Time PCR Experiments (MIQE) checklist is provided in Table S2.

#### RNA extraction

Leaf samples were collected 24 h after 0X (mock) or 50X thiamin treatment from the varieties Snowden and Clearwater Russet on 18 July, 2024 and immediately flash frozen in liquid nitrogen then stored at −80 °C until analysis. For four biological replicates, total nucleic acids were extracted from ∼0.1 g of frozen and ground leaf tissue in 800 µl of extraction buffer containing 0.1 M Tris–HCl, pH 8.0, 0.05 M EDTA, 10 mM *β*-mercaptoethanol, and 0.5 M NaCl (Dellaporta, Wood & Hicks, 1983). All centrifuging steps were set to 17,000 x g and 4 °C. After addition of 70 µl of 10% SDS, samples were incubated for 30 min at 65 °C in a water bath. One hundred µl of 5 M potassium acetate were then added, and samples were incubated on ice for 15 min. After centrifugation for 25 min, 800 µl of supernatant were transferred to a new tube, 300 µl of cold isopropanol was added, and samples were incubated on ice for 5 min. After centrifugation for 20 min, the supernatant was decanted, and the pellet was allowed to dry before resuspension in 100 µl of DNase/RNase free water. RNAs were then precipitated overnight at 4 °C by adding 100 µl of 4 M LiCl to each tube. Samples were then centrifuged for 30 min, the supernatant was discarded, and the pellet was washed in 200 µl of ice cold 70% ethanol. After centrifugation for 15 min, the supernatant was decanted, the pellet was allowed to dry and then was resuspended in 100 µl of DNase/RNase free water. Residual DNA was removed using the DNase Treatment & Removal kit (Invitrogen, Waltham, MA, USA) according to the manufacturer’s recommendations. The absence of genomic DNA in RNA extracts was verified by running qPCR on RNA samples.

#### cDNA synthesis

RNA concentrations were measured with a nanodrop (NanoDrop OneC, Thermo Fisher Scientific, Waltham, MA, USA), and RNA integrity was verified by running samples on 1% agarose gels. RNA integrity checks resulted in the removal of one biological replicate each from 0X and 50X thiamin-treated Snowden, and one biological replicate from 0X-treated Clearwater Russet. cDNAs were synthesized from 1 µg of DNase-treated RNA. RNAs were denatured at 70 °C for 10 min in the presence of a dT18 oligonucleotide (25 µM), in a total volume of 15.75 µl, then samples were kept on ice. After denaturation, 2 µl of 10x RT buffer, 2 µl of 10 mM dNTPs, and 0.25 µl of M-MuLV reverse transcriptase (RT) enzyme (200 U µl^−1^) (New England Biolabs, Ipswich, MA) were added to each sample. Samples were incubated at 42 °C for 60 min. Negative RT controls were prepared by replacing the enzyme with DNase/RNase free water. Samples were then stored at −20 °C.

#### Primer design

Sequence accessions used for primer design for *THIC*, *THI1* and *18S rRNA* are Soltu.DM.06G003240, Soltu.DM.07G025600, and X67238, respectively. All primers were designed in the NCBI Primer Designing Tool. Specificity was checked using default primer specificity parameters using the Refseq mRNA database for *Solanum tuberosum*. Three sets of primers were designed to detect and quantify mRNA *THIC* variants, *i.e.,* intron-retained (IR) variant, intron-spliced (IS) variant, and total *THIC* mRNAs. For the IR variant, the forward primer 5′-AGTGATCACAGCTCCATCGG-3′ (Table S3) is located in an exon in the 3′ untranslated region (UTR), and the reverse primer 5′-TGAACAAGGCTGTTGTCTCAGT-3′ (Table S3) is located in the intron that gets spliced under high thiamin diphosphate levels (Wachter, 2010). The amplicon generated is 116-bp long. For the IS variant, the forward primer 5′-AAGGAGGCGGTAGTAGGAGC-3′ (Table S3) and the reverse primer 5′-CCCGTTCAGGTTCAAAGGGA-3′ (Table S3) are both located in exons in the 3′ UTR on each side of the alternatively spliced intron. The amplicon generated is 89-bp long. For total *THIC* mRNAs, the forward primer 5′-GCGGTGAGATCTACTTGCCA-3′ (Table S3) is located in an exon in the coding sequence and the reverse primer 5′-TCCTACTACCGCCTCCTTGA-3′ (Table S3) is located in an exon in the 3′UTR. The amplicon generated is 135-bp long. For *THI1*, the forward primer 5′-AACCCTGATGTTCAGGTGGC-3′ (Table S3) and the reverse primer 5′-ACGTAGTGGTCTTGCTCGT-3′ (Table S3) are both located in exons in the coding sequence to generate a 159-bp amplicon. For *18S rRNA*, primers were previously described (Nicot et al., 2005). The forward primer 5′-GGGCATTCGTATTTCATAGTCAGAG-3′ (Table S3) and the reverse primer 5′-CGGTTCTTGATTAATGAAAACATCCT-3′ (Table S3) are both located in exons to generate a 101-bp amplicon.

#### Quantitative PCR

Quantitative PCRs were run on a Stratagene Mx3005P (Thermo Fisher Scientific, Waltham, MA, USA) controlled by MxPro-Mx3005p v4.10. Reactions were performed in 96-well PCR plates using the Brilliant III SYBR Green qPCR Master Mix (Thermo Fisher Scientific). Samples were run in either technical duplicates or triplicates. Each well contained 10 µl of 2X SYBR Green Master mix, 0.3 µl of reference dye (1:500), 2 µl each of 1.5 µM forward and reverse primers, 2 µl of cDNA diluted 10 times, and 3.7 µl DNase/RNase free water for a total volume of 20 µl. PCR cycles had an initial denaturation step of 3 min at 95 °C, followed by 44 two-step amplification cycles of 10 s at 95 °C then 20 s at 60 °C. A final three-step melting cycle of 1 min at 95 °C, 30 s at 55 °C, and 30 s at 95 °C was used to check for specificity. To determine PCR efficiency, serial dilutions of cDNAs from 1:10 to 1:500 were used in qPCR reactions and Ct values were plotted against the log_10_ of dilution factor. PCR efficiency was calculated using the formula: ((10^(1/Slope)^)−1) * 100. PCR efficiencies, R^2^ of slopes, and Cq variation at lower limit are indicated in Table S3. Linear dynamic range was from 1:10 to 1:200. Intra-assay variation based on Ct values of technical replicates was <1% (Table S4). Outliers were identified as different from other technical replicates by more than 2 Ct values and were omitted from calculations. Relative expression and fold changes were calculated using the *18S rRNA* as a reference gene using established methods (Schmittgen & Livak, 2008). Relative expression was calculated using 2^−ΔCt^ for each sample. A two-tailed, equal variance Student’s *t*-test was performed in Microsoft Excel to determine statistical significance in relative expression between 0X and 50X treatments. Fold change was calculated using 2^−ΔΔCt^, using the average ΔCt of each treatment.

### Soil microbial respiration

During harvest of the spring/summer 2024 trial of the Snowden variety, soils (bulk + rhizosphere + tar) from pots within the same group were pooled and sieved (six mm) to remove plant debris. Soil from pots that had rotten tubers were omitted. This led to 16 samples (six samples for each of the “no pasteurization—no thiamin” and “no pasteurization—20X thiamin” treatments (eight made of three pots, three made of two pots, and one made of one pot) and four samples of the “no pasteurization—50X thiamin” treatment (three made of two pots, one made of one pot). Soil was immediately stored at 4 °C. Aliquots were also stored at −80 °C for soil metagenome sequencing (see below). A ∼700-ml soil sample of each group was then sent to the Oregon State University Soil Health Laboratory for determination of pH, electroconductivity, and microbial respiration.

### Soil metagenome sequencing

Genomic DNA from soil samples “no pasteurization –no thiamin” and “no pasteurization –20X thiamin” treatments collected as above and stored at −80 °C was extracted using the E.Z.N.A. soil DNA kit according to the manufacturer’s instructions (Omega Bio-tek, Norcross, GA, USA). We chose the 20X treatment because microbial respiration increased the most under this treatment (see Results). Six samples of each treatment were pooled in equal amount before extraction. Samples were dried in a speed vacuum. The Rapid Barcoding Kit 96 V14 (SQK-RBK114.96) was used to prepare multiplexed libraries for Oxford Nanopore sequencing. Libraries were sequenced on a PromethION flow cell on an Oxford Nanopore P2 solo sequencer (Oxford Nanopore Technologies, Oxford, UK). Dorado v.0.7.0 with the v5.0.0 SUP model was used to basecall reads (Oxford Nanopore Technologies, 2025). Kraken2 v.2.1.3 with the parameters “–use-names –memory-mapping—threads 24” and the Standard database (downloaded on May 25, 2024) was used to classify the taxonomy of each sequencing read (Wood, Lu & Langmead, 2019). Bracken v.2.7 with the parameters “-r 300 -l G -t 10” was used to convert read counts to estimates of taxonomic abundance for each sample (Lu et al., 2017). The Pavian webserver was used to generate Sankey plots of kraken output (Breitwieser & Salzberg, 2016). Bracken_plot was used to generate stacked bar plots from bracken output (Vill, 2023).

### Metabolite analysis by gas chromatography coupled with mass spectrometry

Tuber metabolites were analyzed by GC-MS using previously published methods (Bvindi et al., 2023). Briefly, 50 mg of dried tuber tissue was extracted in a solution of water:methanol:chloroform (1:2.5:1) with ribitol (40 µg ml^−1^) as an internal standard. After removal of cellular debris, extracts were phase separated to isolate aqueous metabolites, then 50 µl aliquots were frozen and lyophilized to dryness. Dried samples were stored at −80 °C until derivatization. A no-tissue extraction was included as a reagent blank.

Prior to GC-MS analysis, dried samples were derivatized in a two-step process: samples were resuspended in 20 µl methoxamine HCl (30 mg ml^−1^) dissolved in pyridine and incubated at 37 °C for 90 min with shaking, then 40 µl N-methyl-N-trimethylsilyl-trifluoroacetamide with 1% chlorotrimethylsilane was added to each sample followed by a 30 min incubation at 37 °C with shaking. One µl of each sample was injected with a 4:1 split into an Agilent 7890B/5977B instrument equipped with a 30 m plus 10 m Duraguard 0.25 mm x 0.25 µm DB-5MS+DG Agilent column. The oven temperature was kept at 60 °C for 1 min, then ramped to 300 °C at a rate of 10 °C/min and held at 300 °C for 10 min. Analytes were detected in EI mode scanning from 50 m/z to 600 m/z. The sample injection order was randomized and each sample was injected in duplicate. The reagent blank was injected after every three samples to control for potential carry over.

Mass spectrum analysis was performed using AMDIS (Davies, 1998) and the Agilent Fiehn 2013 GC/MS Metabolomics RTL Library (Kind et al., 2009) for automated component identification. Statistical analyses were performed with MetaboAnalyst 6.0 (https://www.metaboanalyst.ca/, accessed on 21 May 2025) using default processing settings and data filtering. Features with ≥2 missing values were removed and variables with missing values were excluded. A 5% variance filter was applied based on the interquartile range. Then, the data were normalized to the reference feature ribitol.

### Statistical analysis

For the winter/spring 2024 experiment, several main stems broke weeks before harvest. Therefore, these plants were eliminated from yield data analyses, resulting in experimental units of one, two or three plants. For the spring/summer 2024 experiment, there were pots that contained rotten tubers. In this case, all the tubers from those pots were discarded and not used in yield data analysis, resulting in experimental units of one, two or three plants. The occurrence of broken stems or rotten tubers was randomly distributed between treatments, so the potential bias introduced by these exclusions is not significant. For the determination of statistical differences between soil treatments, we performed Analysis of Variance (ANOVA) with a Tukey’s HSD test or Student’s *t*-test (one-tailed). For tuber thiamin-content data, we checked the normality of residuals using a Shapiro–Wilk normality test and the homogeneity of variances using a Levene’s test. If normality was not met, we applied an inverse transformation on thiamin concentrations.

## Results

### Soil thiamin supplementation had no effect on yield under optimal growth greenhouse conditions

To test the effect of soil thiamin supplementation on potato tuber yield, we first grew plants of the variety Clearwater Russet in pasteurized and non-pasteurized soils supplemented with three different concentrations of thiamin. Pasteurization was chosen as a soft method to kill the soil microbiome without affecting soil physical and chemical properties. The goal of pasteurization was to obtain a baseline with no soil microbes-produced thiamin. The lowest amount of thiamin added to soil (1X = 5 mg kg^−1^ soil) was based on concentrations normally found in soils (Schmidhalter et al., 1994) although wide ranges of concentrations have been reported (Kononova, 1966; Mozafar, 1994). Soil pasteurization and thiamin supplementation had no effect on total tuber yield, number of tubers per plant and tuber weight distribution of Clearwater Russet potatoes grown in spring/summer 2023 (Table 2).

**Table 2:** Total tuber yield (grams per plant). Identical letters within a column indicate that there was no significant difference between treatments as determined by ANOVA (*P* = 0.05). For the spring/summer 2023 season, data are means ± SE of nine to 11 biological replicates (one replicate = one plant). For the winter/spring 2024 season, data are means ±  SE of three to four replicates (one replicate = two to three plants). For the spring/summer 2024, data are means ± SE of four to six replicates (one replicate = one to three plants).

	Spring/Summer 2023	Winter/Spring 2024	Spring/Summer 2024	
	Clearwater Russet	Russet Norkotah	Clearwater Russet	Snowden
Not pasteurized—no thiamin	407 ± 40^a^	255 ± 45^a^	408 ± 58^a^	658 ± 86^a^
Not pasteurized—20X thiamin	n.a.	226 ± 55^a^	390 ± 49^a^	771 ± 56^a^
Not pasteurized—50X thiamin	n.a.	271 ± 52^a^	369 ± 81^a^	694 ± 158^a^
Pasteurized—no thiamin	404 ± 31^a^	201 ± 30^a^	n.a.	n.a.
Pasteurized—1X thiamin	454 ± 43^a^	n.a.	n.a.	n.a.
Pasteurized—5X thiamin	394 ± 49^a^	n.a.	n.a.	n.a.
Pasteurized—20X thiamin	478 ± 39^a^	268 ± 28^a^	n.a.	n.a.
Pasteurized—50X thiamin	n.a.	264 ± 35^a^	n.a.	n.a.

**Notes.**

n.a., not applicable.

In winter/spring 2024, Russet Norkotah potatoes were grown on non-pasteurized and pasteurized Sunshine Mix#4, which was supplemented with two concentrations of thiamin (20X and 50X). We used a higher concentration (50X) than the previous season based on the lack of effect on tuber yield at the highest concentration (20X) used in the previous experiment. Total tuber yield, the number of tubers per plant and tuber weight distribution did not change significantly between treatments (Table 2, Figs. S2 and S3). Soil samples analyzed before and after pasteurization before planting indicated a modest 30–35% decrease in microbial respiration (Table S5), suggesting that pasteurization was not effective at killing all soil microbes. Therefore, pasteurization was not used in our next experiments.

In spring/summer 2024, we grew two potato varieties in Adkins soil collected from the lower Columbia Basin in Oregon. This soil type is representative of the type of soil where potatoes are grown commercially in the Pacific Northwest. Soil thiamin supplementation up to 50X had no effect on total tuber yield, the number of tubers per plant and tuber weight distribution of either variety (Table 2, Figs. S2 and S3). The highest thiamin concentration decreased specific gravity in Snowden, but not in Clearwater Russet (Fig. S4).

### Thiamin accumulated in plants grown on soil supplemented with thiamin

**Figure 1:**
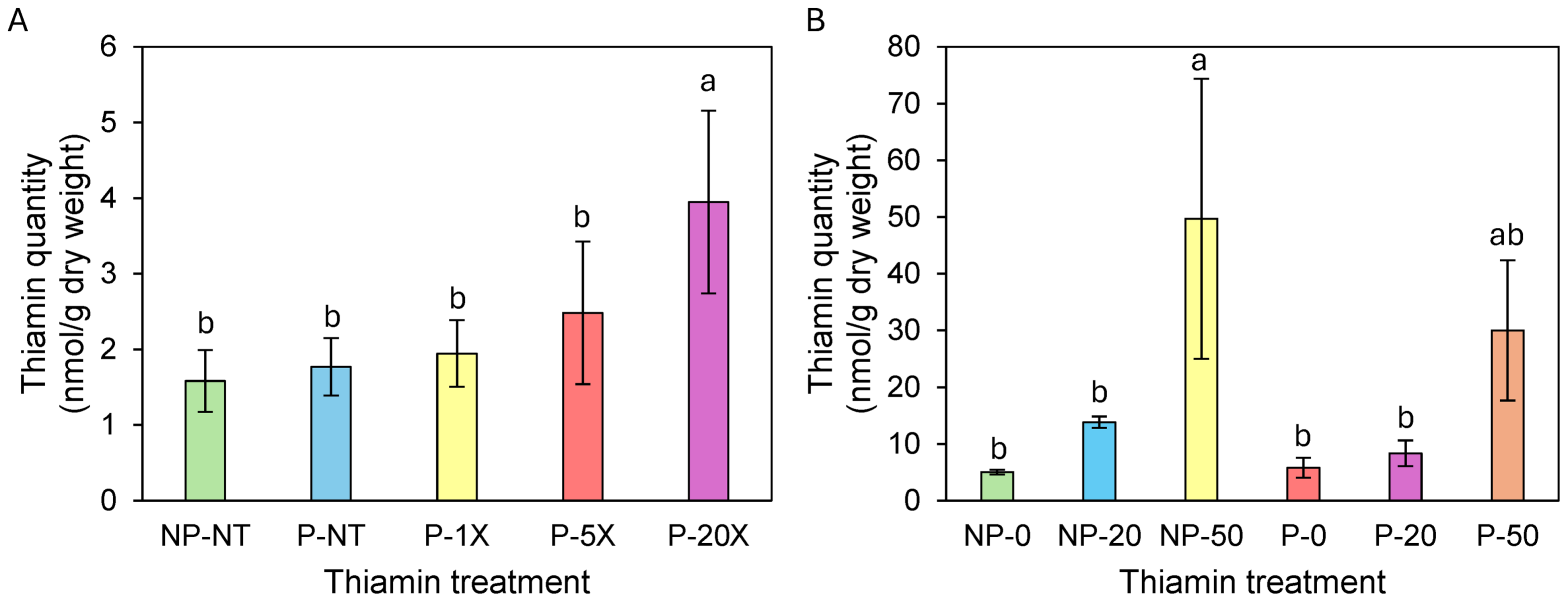
Thiamin content in Clearwater Russet (A) and Russet Norkotah (B) tubers. Identical letters indicate that there were no significant differences between samples as determined by ANOVA (P¡=0.05). NP, soil not pasteurized; P, pasteurized soil; NT, no thiamin.

In spring/summer 2023, there was no significant accumulation of thiamin in tubers from Clearwater Russet plants grown on soil supplemented with 1X and 5X thiamin (Fig. 1A). However, thiamin content was 3.3-fold higher in tubers from plants grown on 20X thiamin compared to tubers from plants that were not supplemented with thiamin (Fig. 1A). In winter/spring 2024, thiamin content in tubers of Russet Norkotah grown on soil supplemented with 20X thiamin did not increase compared to control plants. However, thiamin tuber content increased when plants were grown on soil supplemented with 50X thiamin (Fig. 1B). In spring/summer 2024, tubers of Clearwater Russet and Snowden accumulated 2.3 and 1.2 more thiamin, respectively, when plants were grown on 20X thiamin, and 5.8 and 2.8 times more thiamin, respectively, when plants were grown on 50X thiamin (Table 3).

**Table 3:** Total thiamin concentrations (nmol g^−1^ dry weight) in different organs of potato plants grown on soil supplemented with thiamin during the spring/summer 2024 season. Treatments with identical letters within a column indicate no significant difference between treatments as determined by ANOVA (tubers and roots) or Student’s *t* test (leaves and stems) (*P* = 0.05). Data are means ± SE of three or four replicates (one replicate = one experimental unit).

	Clearwater Russet				Snowden			
	Tubers	Leaves	Stems	Roots	Tubers	Leaves	Stems	Roots
No thiamin	8.9 ± 0.5^a^	3.9 ± 1.7^a^	21.9 ± 10.4^a^	110.1 ± 10.5^a^	7.6 ± 0.7^a^	3.8 ± 2.4^a^	10.3 ± 3.5^a^	130.7 ± 1.6^a^
20X thiamin	20.9 ± 4.1^a^	n.d.^2^	n.d.	2,106.5 ± 957.7^ab^	9.1 ± 0.7^ab^	n.d.	n.d.	2,877.5 ± 1009.2^ab^
50X thiamin	51.4 ± 11.1^b^	8.7 ± 3.0^a^	34.7 ± 5.0^a^	6,430.7 ± 1,803.2^b^	21.4 ± 5.4^b^	15.6 ± 3.8^b^	31.9 ± 6.0^b^	6,862.0 ± 1,965.2^b^
% increase^1^	477	123	58	5,745	182	310	209	5,178

**Notes.**

1 Increase between no thiamin and 50X thiamin.

2 n.d., not determined.

**Figure 2:**
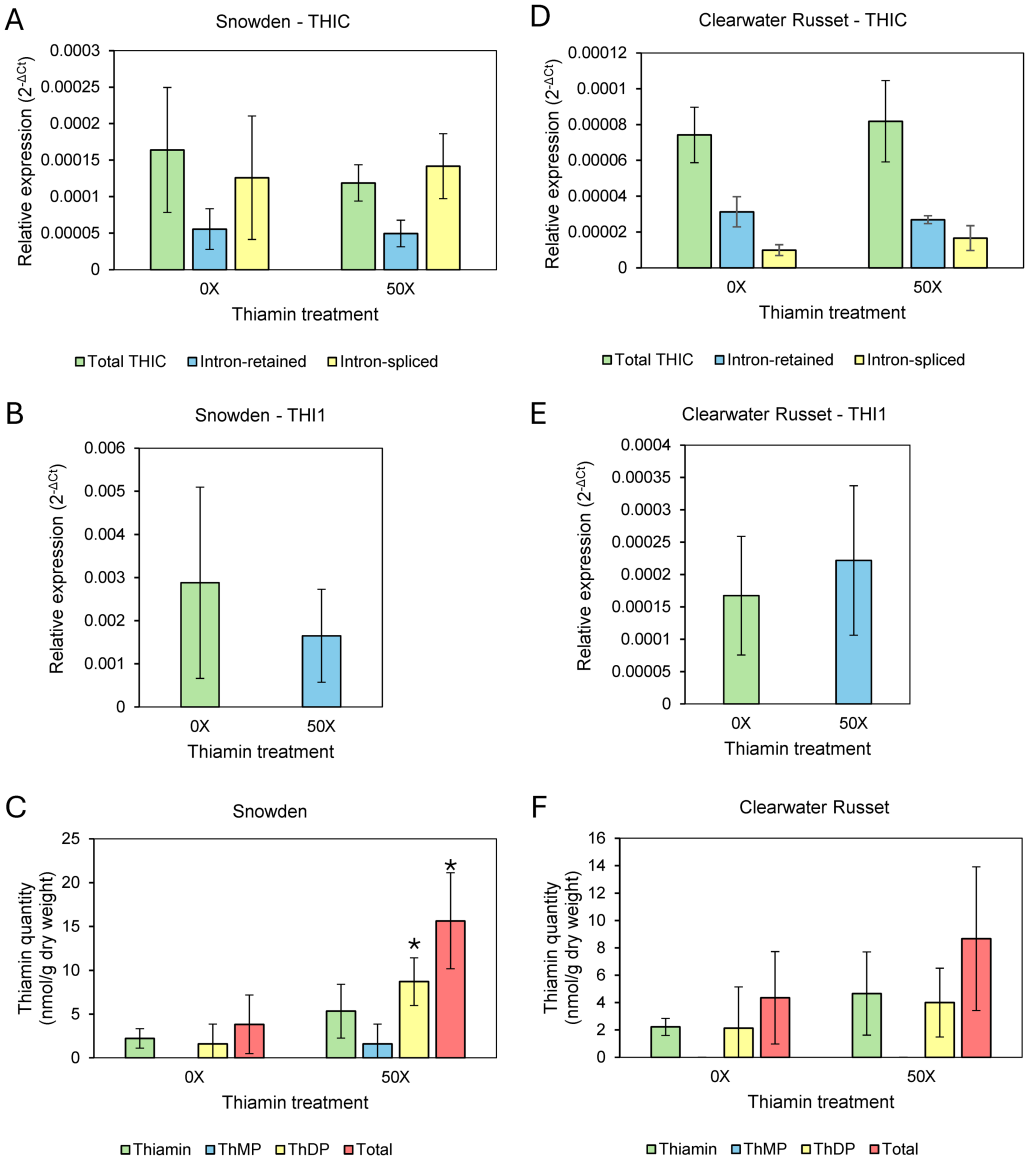
Relative expression of thiamin biosynthesis genes and thiamin quantities in leaves of 0X and 50X thiamin-treated plants. (A, D) Expression of THIC isoforms in Snowden and Clearwater Russet, (B, E) expression of THI1 in Snowden and Clearwater Russet , and (C, F) quantity of thiamin species (ThMP = thiamin monophosphate, ThDP = thiamin diphosphate) in the leaves of Snowden and Clearwater Russet plants. * Indicates significant differences (*p* < 0.05) between thiamin treatments as determined by one-tailed Student’s *t*-test. Relative expression calculated using 18S as a reference gene, calculated according to established methods (Schmittgen & Livak, 2008).

Roots of Clearwater Russet and Snowden accumulated about 20 and over 50 times more thiamin when plants were grown on 20X and 50X thiamin, respectively, compared to plants that were not supplemented with thiamin (Table 3). Stems of Snowden accumulated three times more thiamin when plants were grown on soil supplemented with 50X thiamin, while thiamin content of stems of Clearwater Russet was not statistically different between plants grown with or without thiamin supplementation (Table 3). Thiamin did not accumulate significantly in leaves of Clearwater Russet in plants grown with thiamin supplementation (Table 3, Fig. 2F). However, total thiamin content significantly increased in leaves of Snowden plants grown with thiamin supplementation (Table 3, Fig. 2C).

### Expression of thiamin biosynthesis genes THI1 and THIC

Our initial hypothesis was that thiamin applied to the root-zone of plants would be transported to leaves and that concentrations of thiamin, as well as the phosphorylated thiamin derivative ThDP, would increase in leaves, resulting in lowered thiamin biosynthesis through the ThDP riboswitch present in the *THIC* gene (Bocobza et al., 2007; Bocobza & Aharoni, 2008). Thiamin quantification showed that thiamin concentrations increased in leaves of Snowden plants grown on thiamin-supplemented soil but not in leaves of Clearwater Russet (Table 3, Figs. 2C & 2F). Therefore, we analyzed the expression of *THIC* and *THI1* in leaves of both varieties. Gene expression analysis between 0X and 50X thiamin-treated plants grown in spring/summer 2024 showed that expression of *THI1* and *THIC* were not significantly altered by soil application of thiamin (Fig. 2). Analysis of intron retained and intron spliced isoforms of *THIC* were also not statistically different between treatments (Figs. 2A & 2D).

### Primary metabolites in tubers

**Figure 3:**
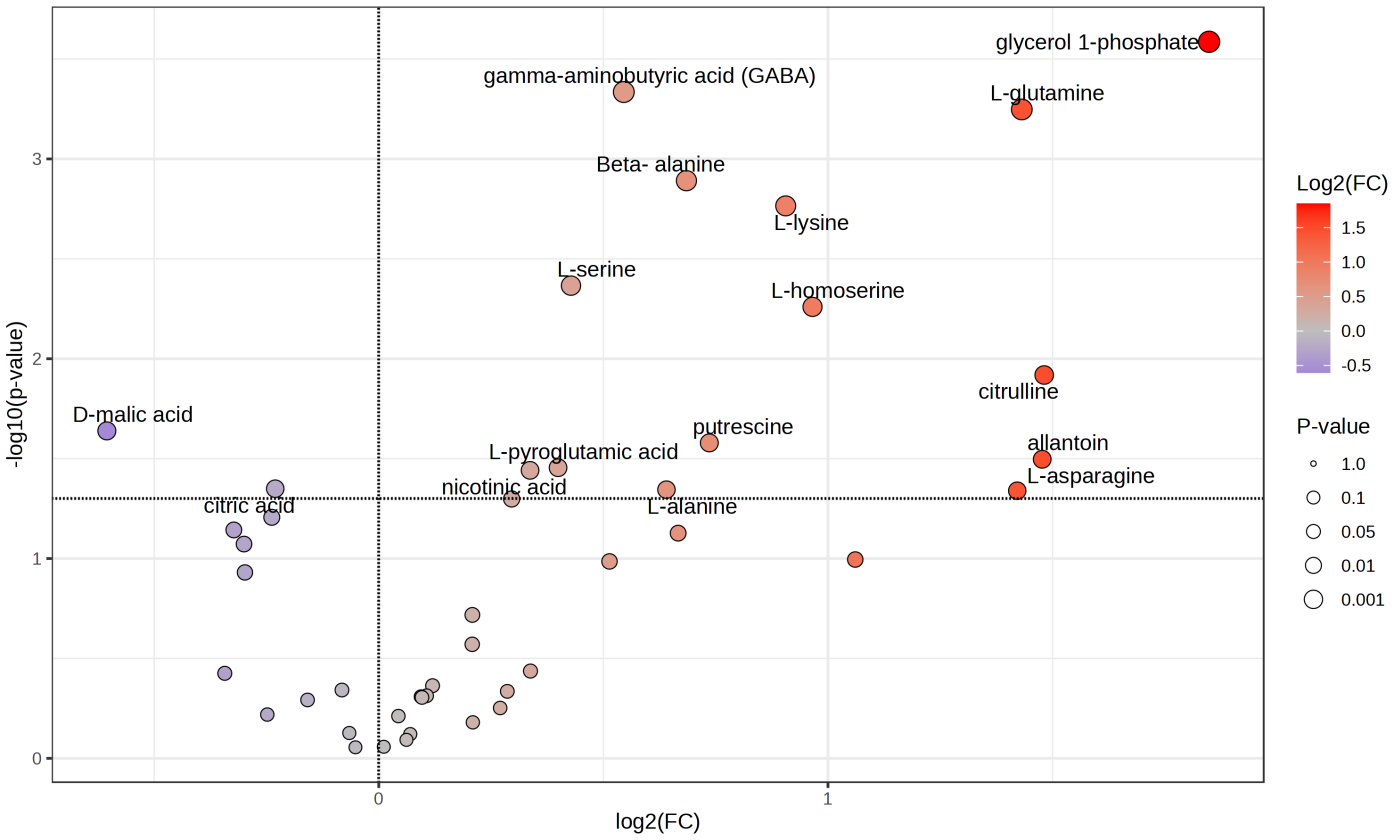
Volcano plot of tuber metabolites. Labeled compounds are statistically significant (*p* < 0.05) according to Student’s *t*-test.

**Figure 4:**
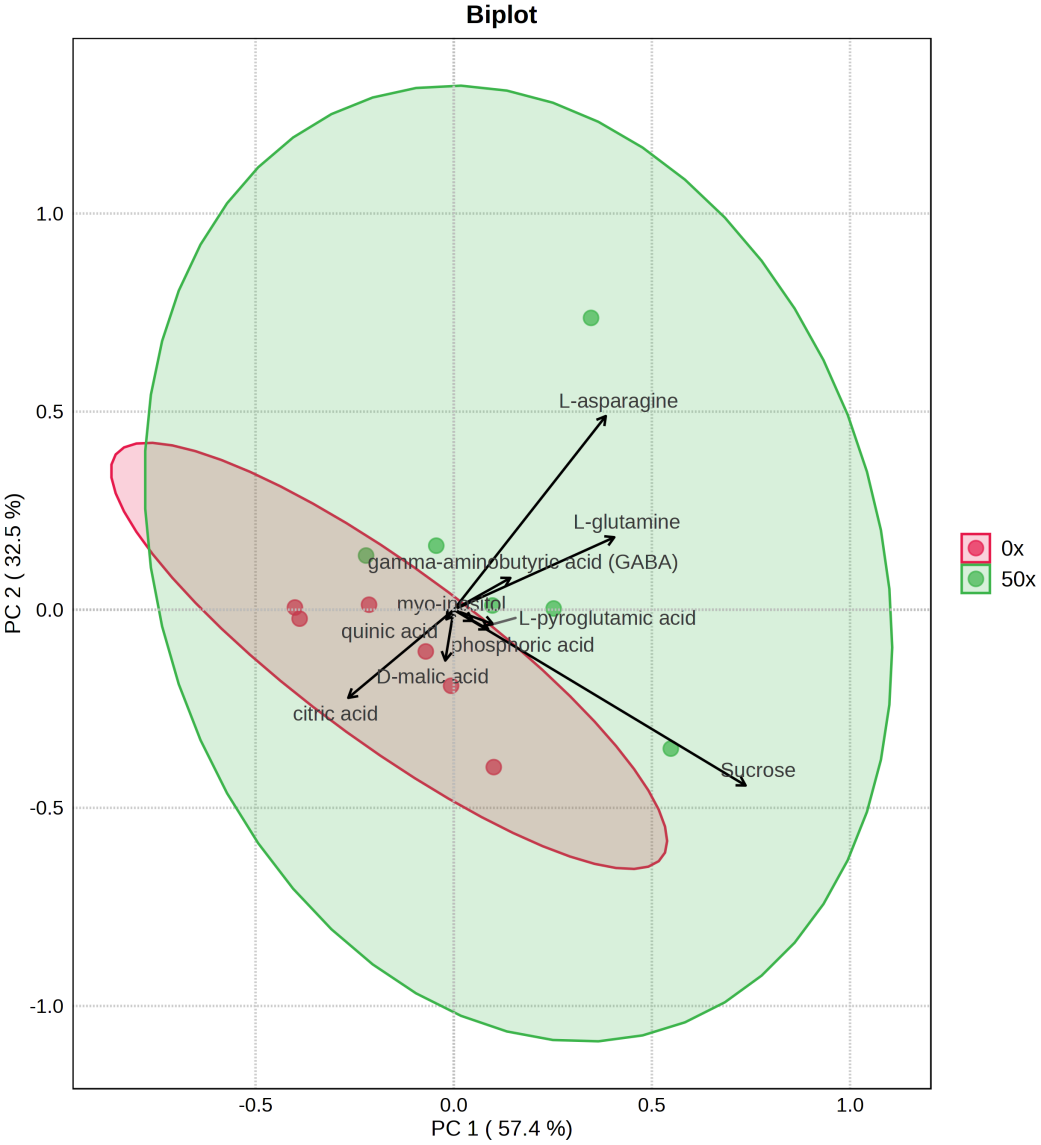
PCA biplot of tuber metabolites with top 10 features displayed.

Because tubers from plants grown on soil supplemented with 50X thiamin accumulated thiamin, we were interested in the potential effect of thiamin overaccumulation on tuber metabolism. Therefore, we used GC-MS to analyze metabolites in Clearwater Russet tubers collected from plants grown with no or 50X thiamin. Of 112 compounds detected, 48 passed filtering criteria, *i.e.,* less than two missing values. Two were statistically reduced and 14 were statistically increased with 50X thiamin. Notably, the abundance of multiple amino acids, such as *β*-alanine, lysine, glutamine, and asparagine, were increased by 50X thiamin treatment (Fig. 3). However, in a principal component analysis, the 0X and 50X treatments did not group separately (Fig. 4), suggesting limited systemic metabolic reprogramming.

### Soil thiamin supplementation increased microbial respiration and altered microbiome composition

**Figure 5:**
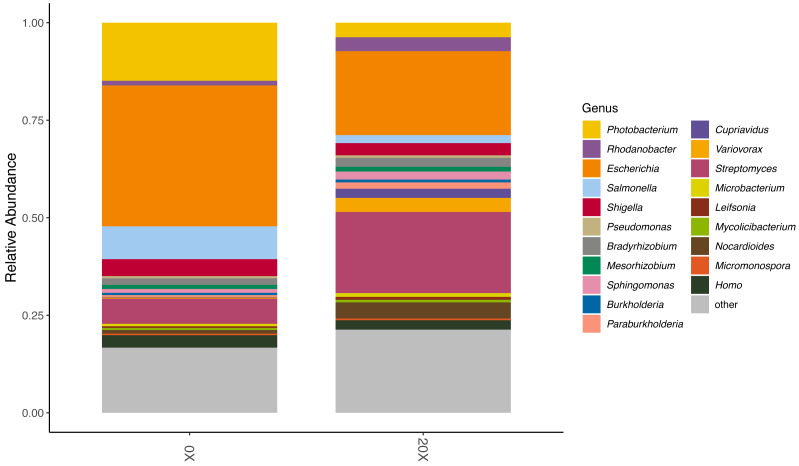
Relative abundance of organisms in soil metagenome samples. The relative abundance of the top 20 most abundant genera in the 0X and 20X thiamin-supplemented samples are represented by colored blocks. The relative abundance of all other taxa is gray.

We assessed the effect of thiamin supplementation on soil chemical and biological characteristics from the Snowden experiment. Thiamin supplementation of Adkins soil significantly increased microbial CO_2_ respiration at 20X and 50X (Table 4). At 50X, pH also became significantly more acidic. There was no change in electroconductivity and active carbon content. Because of the increased respiration in soil amended with thiamin, we performed long-read metagenome sequencing to assess possible changes in the microbiota composition. Nanopore sequencing of total DNA from 0X and 20X thiamin-supplemented soil samples resulted in 555,482 and 723,492 reads, respectively, with mean read lengths of 403 and 501 bp. Kraken successfully classified 301,385 and 404,877 reads from the 0X and 20X samples, respectively, or 54.3% and 56.0% of total reads. Kraken analysis revealed that the microbial community of potato soil was diverse (Figs. S5 and S6). The potato soil microbiota was largely dominated by Actinomycetota and Pseudomonadota. The most abundant microbial taxa were various species of *Streptomyces* and *Photobacterium* (Figs. S5 and S6). Comparing the 0X and 20X samples revealed that the relative abundance of *Photobacterium* was lower in the 20X sample relative to the 0X sample (Fig. 5). Inversely, the relative abundance of *Streptomyces* was greater in the 20X sample than the 0X sample. The relative abundance of *Rhodanobacter* also increased in the 20X sample *versus* the 0X sample. Most other taxa maintained similar levels of relative abundance in both treatments.

**Table 4:** Soil pH, electroconductivity, active carbon, and microbial respiration after harvest of Snowden potatoes. Identical letters indicate that there was no significant difference between treatments within column as determined by ANOVA (*P* = 0.05). Data are means ± SE of six (0X and 20X) or four (50X) replicates (one replicate = one to three pooled pots).

Treatment	pH	EC	Active C	CO_2_ respiration 24 h	CO_2_ respiration 96 h
0X	5.43 ± 0.08^a^	0.44 ± 0.04^a^	83.8 ± 1.8^a^	5.7 ± 0.2^a^	4.5 ± 0.3^a^
20X	5.19 ± 0.06^ab^	0.62 ± 0.06^a^	92.0 ± 5.6^a^	9.2 ± 0.4^b^	7.7 ± 0.4^b^
50X	4.99 ± 0.03^b^	0.68 ± 0.14^a^	85.0 ± 8.5^a^	8.0 ± 0.4^b^	7.0 ± 0.4^b^

## Discussion

In this study, we investigated the effect of soil thiamin supplementation on potato tuber yield and thiamin content. Our main finding is that exogenously supplied thiamin has the potential to biofortify potato tubers. Although we did not observe changes in yield, there are several limitations in our experiments that may have prevented us from detecting an effect. We discuss these limitations below.

Our study indicates that providing plants with higher thiamin concentrations in the root zone might be a viable strategy for thiamin biofortification of potato and possibly other crops. Indeed, tubers harvested from plants grown on soil supplemented with thiamin accumulated up to 51.4 nmol g^−1^ DW, approximately ∼6 times more than tubers harvested from plants grown on non-supplemented soil. Assuming 20% tuber dry matter and an 86% retention of thiamin after cooking (Goyer & Haynes, 2011), an average size (150 g) thiamin-biofortified potato would provide 0.45 mg of thiamin, or ∼37% of a healthy male adult daily need. For reference, in Europe, North America, and Asia, per capita yearly consumption of potato between 2017 and 2021 reached 76 kg, 53.2 kg, and 31 kg, respectively (FAO, 2023). Another positive aspect of thiamin-biofortified tubers is that changes in the overall metabolome seemed minimal, suggesting that it should be possible to change potato tuber nutritional value specifically for thiamin. In addition, starch content is an essential quality determinant for processing potatoes into fried products. Our observation that starch content of thiamin-biofortified tubers did not change suggests that these tubers should be suitable for processing.

The level of thiamin biofortification achieved in this study was based on weekly soil supplementation with high thiamin concentrations throughout the growth period of the plants. We chose to replenish soil on a weekly basis based on the assumption that thiamin would be degraded and/or metabolized in the soil or that all the supplied thiamin would be taken up by plants relatively quickly. However, it remains unclear what proportion of applied thiamin was used by the plants or degraded in the soil. Our attempts to quantify thiamin that remained in the soil failed because of low recovery of thiamin using a water extraction method. It is not clear, therefore, whether high concentrations and frequency of application are necessary to increase tuber thiamin content. Application of thiamin at key growth stages might achieve similar biofortification levels, *e.g.*, shortly before harvest. It is also not clear whether reaching soil thiamin concentrations necessary for tuber thiamin enrichment by application of organic fertilizers is achievable. Further research will be necessary to answer these questions.

Our study also indicated that soil thiamin supplementation did not increase tuber yield. However, it is important to note several limitations in our experiments. First, the small sample size of our experiments may not have detected a predicted maximum yield increase of 4.2% (Hanson et al., 2018). The statistical power achieved for yield comparisons (*p* < 0.05) ranged between ∼6% and ∼45% depending on the experiment and the treatment chosen for the determination of variance. Second, we did not measure biomass of green tissues and roots and cannot rule out that soil thiamin supplementation may have influenced biomass accumulation in these parts of the plant. Third, we obtained our data within a specific environment, *i.e*., optimal growth conditions in a greenhouse. Therefore, it is not yet clear whether field-scale trials under stress conditions (*e.g.*, drought, salinity) would yield similar results. Under stress conditions, plants need to produce more thiamin due to increased thiamin degradation (Hanson et al., 2016). At the same time, THI1 and THIC turnover rates likely increase (Hanson et al., 2018). Therefore, the energy spent towards thiamin biosynthesis increases under stress. Interestingly, exogenous supply of thiamin in the growth medium can enhance plant tolerance to abiotic stress, *i.e.,* salt, osmotic, cold (Al-Hakimi & Hamada, 2001; Kaya et al., 2015; Li et al., 2022; Sayed & Gadallah, 2002; Tunc-Ozdemir et al., 2009). Further studies should investigate the effect of soil thiamin supplementation on potato tuber yield in large scale experiments and under stress conditions.

Besides limitations inherent to experimental conditions, our data suggest that factors intrinsic to potato plant physiology might explain the absence of yield increase. We hypothesized that leaf thiamin content is the key determinant of yield based on the following reasons. First, one study has shown that direct foliar thiamin application increased potato tuber yield (Iijima, 1960), and other studies have shown that thiamin applied to leaves can accumulate to high levels in treated leaves (Mozafar & Oertli, 1992; Mozafar & Oertli, 1993). Second, although sink tissues, *i.e.,* roots and possibly tubers, have the capability to synthesize thiamin *de novo*, they are not thiamin self-sufficient and must import it from source tissues, *i.e.,* mature leaves where thiamin production is the highest. Therefore, supplementing thiamin to leaves has the highest energy-saving potential. However, our data indicate that transport of thiamin from roots to above-ground tissues was severely restricted. Indeed, thiamin concentrations in roots increased over 50 times under high thiamin treatment and proportionally to the amounts of thiamin added to soil (thiamin concentrations are 2.4- to 3.0-fold higher in 50X treatment compared to 20X). This indicates that thiamin uptake by roots most likely occurred passively by diffusion and that the levels of thiamin that accumulate in root tissues are solely dependent on thiamin concentrations in the surrounding soil. Thiamin concentrations in stems and leaves, however, did not increase or increased only modestly in our experiments. These results are in agreement with studies in soybean seedlings that have reported modest levels of thiamin accumulation in leaves of plants fed thiamin through the roots (Mozafar & Oertli, 1992; Mozafar & Oertli, 1993), although partitioning depended on thiamin concentrations in the media. These studies also indicated that thiamin uptake and transport do not require metabolic energy (Mozafar & Oertli, 1992; Mozafar & Oertli, 1993). Therefore, our results point to an inefficient transport of thiamin from roots to above-ground tissues, especially leaves, which could have hindered yield improvement.

Finally, soil thiamin application triggered shifts in soil microbial communities. The most striking change was the increase in the relative abundance of *Streptomyces* species. The *Streptomyces* genus encompasses many species that are important for agriculture. They play an important role in soil organic matter catabolism and have biocontrol and plant growth-promoting properties (Olanrewaju & Babalola, 2019). We also observed a relative enrichment in *Rhodanobacter* species, which play a crucial role in acidic denitrification (Van Den Heuvel et al., 2010). Because *Rhodanobacter* species thrive in low pH, we cannot rule out that the slight acidification of the soil after thiamin application might have contributed to the relative enrichment in these species. It will be interesting to investigate whether soil application of thiamin-rich sources like green manures produce the same beneficial effect on the soil microbiome.

## Conclusions

This study shows that soil thiamin supplementation might be a viable strategy to increase the thiamin content of potato tubers to levels that might be difficult to attain by traditional breeding (Goyer & Haynes, 2011; Goyer & Sweek, 2011). Although the application of commercial synthetic thiamin is likely not economically sustainable, growers could apply organic fertilizers like green manures, which are rich sources of thiamin. Future work could also investigate engineering of the soil microbiome to increase soil thiamin concentrations. Under our experimental conditions, soil thiamin supplementation did not improve yield. However, the sample size may have been too small to detect modest expected yield increases (<5%). Therefore, field-scale experiments will be necessary to truly assess the effect of soil thiamin supplementation on yield. In addition, future work could investigate the effect of soil thiamin supplementation on yield under stress conditions that are known to increase plants’ needs for thiamin, for instance cold and salt (Tunc-Ozdemir et al., 2009). Finally, engineering of an active thiamin uptake in plant roots and transport to above-ground tissues should be investigated to increase soil thiamin use efficiency.

## Supplemental Information

10.7717/peerj.20684/supp-1 Supplemental Information 1 Schematic representation of experimental designs used in spring/summer 2023 (A), winter/spring 2024 (B), and spring/summer 2024 (C) Each color represents a different thiamin treatment. White, “no pasteurization –no thiamin”; blue, “no pasteurization –20X thiamin”; green, “no pasteurization –50X thiamin”; yellow, “pasteurization –no thiamin”; grey, “pasteurization –1X thiamin”; dark grey, “pasteurization –5X thiamin”; orange, “pasteurization –20X thiamin”; red, “pasteurization –50X thiamin”.

10.7717/peerj.20684/supp-2 Supplemental Information 2 Number of tubers per plant There was no significant difference in the number of tubers between thiamin treatments. NP, soil not pasteurized. P, pasteurized soil. Graphs were generated in Graphpad Prism version 10.4.1 for Windows (GraphPad Software, Boston, Massachusetts USA ).

10.7717/peerj.20684/supp-3 Supplemental Information 3 Number of tubers per plant per weight categories There was no significant difference in the distribution of tuber weight between thiamin treatments. NP, soil not pasteurized. P, pasteurized soil.

10.7717/peerj.20684/supp-4 Supplemental Information 4 Specific gravity in Snowden and Clearwater Russet Identical letters indicate that there were no significant differences between samples as determined by ANOVA (*P* < 0.05). Graphs were generated in Graphpad Prism version 10.4.1 for Windows (GraphPad Software, Boston, Massachusetts USA).

10.7717/peerj.20684/supp-5 Supplemental Information 5 Sankey plot of the bacterial microbiome in soil that did not receive thiamin

10.7717/peerj.20684/supp-6 Supplemental Information 6 Sankey plot of the bacterial microbiome found in soil that received 20X thiamin

10.7717/peerj.20684/supp-7 Supplemental Information 7 Soil temperature and water content measured using Terros 12 sensors during the spring/summer growing season 2024 Two and three sensors were used to determine soil temperature and water content in pots with Clearwater Russet (Ports 1 and 2) and Snowden (Ports 1, 2 and 5), respectively. Soil water content calibration was performed according to the manufacturer’s recommendations.

10.7717/peerj.20684/supp-8 Supplemental Information 8 MIQE checklist for authors, reviewers and editors

10.7717/peerj.20684/supp-9 Supplemental Information 9 Primers used in this study The sequence of primers, amplicon size, and efficiency are indicated as well as R square of efficiency slope and Cq variation at lower limit (1:200 dilution).

10.7717/peerj.20684/supp-10 Supplemental Information 10 Raw data

10.7717/peerj.20684/supp-11 Supplemental Information 11 Soil microbial respiration at planting and at harvest Three soil samples per treatment (NP, not pasteurized; P, pasteurized) were analyzed for moisture content, active carbon, CO2 respiration, pH, and electroconductivity (EC).

## Additional Information and Declarations

### Data Availability

The following information was supplied regarding data availability: The raw data are available in the Supplementary File and at SRA: PRJNA1294382, SAMN50092898, and SAMN50092899.

## References

[ref-1] Al-Hakimi AMA, Hamada AM (2001). Counteraction of salinity stress on wheat plants by grain soaking in ascorbic acid, thiamin or sodium salicylate. Biologia Plantarum.

[ref-2] Bocobza S, Adato A, Mandel T, Shapira M, Nudler E, Aharoni A (2007). Riboswitch-dependent gene regulation and its evolution in the plant kingdom. Genes and Development.

[ref-3] Bocobza SE, Aharoni A (2008). Switching the light on plant riboswitches. Trends in Plant Science.

[ref-4] Bonner J, Greene J (1938). Vitamin B1 and the growth of green plants. Botanical Gazette.

[ref-5] Bonner J, Greene J (1939). Further experiments on the relation of vitamin B1 to the growth of green plants. Botanical Gazette.

[ref-6] Breitwieser FP, Salzberg SL (2016). Pavian: interactive analysis of metagenomics data for microbiomics and pathogen identification. BioRxiv.

[ref-7] Bvindi C, Howe K, Wang Y, Mullen RT, Rogan CJ, Anderson JC, Goyer A (2023). Potato non-specific lipid transfer protein StnsLTPI.33 is associated with the production of reactive oxygen species, plant growth, and susceptibility to *Alternaria solani*. Plants.

[ref-8] Chatterjee A, Abeydeera ND, Bale S, Pai PJ, Dorrestein PC, Russell DH, Ealick SE, Begley TP (2011). *Saccharomyces cerevisiae* THI4p is a suicide thiamine thiazole synthase. Nature.

[ref-9] Davies AN (1998). The new Automated Mass Spectrometry Deconvolution and Identification System (AMDIS). Spectroscopy Europe.

[ref-10] Dellaporta SL, Wood J, Hicks JB (1983). A plant DNA minipreparation: version II. Plant Molecular Biology Reporter.

[ref-11] Dong W, Stockwell VO, Goyer A (2015). Enhancement of thiamin content in *Arabidopsis thaliana* by metabolic engineering. Plant and Cell Physiology.

[ref-12] FAO (2023). FAOSTAT. https://www.fao.org/faostat/.

[ref-13] Goyer A (2010). Thiamine in plants: aspects of its metabolism and functions. Phytochemistry.

[ref-14] Goyer A (2017). Thiamin biofortification of crops. Current Opinion in Biotechnology.

[ref-15] Goyer A, Haynes KG (2011). Vitamin B1 content in potato: effect of genotype, tuber enlargement, and storage, and estimation of stability and broad-sense heritability. American Journal of Potato Research.

[ref-16] Goyer A, Sweek K (2011). Genetic diversity of thiamin and folate in primitive cultivated and wild potato (*Solanum*) species. Journal of Agricultural and Food Chemistry.

[ref-17] Hanson AD, Amthor JS, Sun JY, Niehaus TD, Gregory JF, Bruner SD, Ding YS (2018). Redesigning thiamin synthesis: prospects and potential payoffs. Plant Science.

[ref-18] Hanson AD, Beaudoin GA, McCarty DR, Gregory JF (2016). Does abiotic stress cause functional B vitamin deficiency in plants?. Plant Physiology.

[ref-19] Iijima T (1960). Studies on the physiology and utilization of vitamin B1 in some garden crops. Journal of the Faculty of Agriculture Shinshu University.

[ref-20] Kaya C, Ashraf M, Sonmez O, Tuna AL, Polat T, Aydemir S (2015). Exogenous application of thiamin promotes growth and antioxidative defense system at initial phases of development in salt-stressed plants of two maize cultivars differing in salinity tolerance. Acta Physiologiae Plantarum.

[ref-21] Kind T, Wohlgemuth G, Lee do Y, Lu Y, Palazoglu M, Shahbaz S, Fiehn O (2009). FiehnLib: mass spectral and retention index libraries for metabolomics based on quadrupole and time-of-flight gas chromatography/mass spectrometry. Analytical Chemistry.

[ref-22] Kononova MM, Kononova MM (1966). Importance of organic matter in soil formation and fertility. Soil organic matter.

[ref-23] Lemmer H, Nitschke L (1994). Vitamin content of four sludge fractions in the activated sludge wastewater treatment process. Water Research.

[ref-24] Li W, Mi X, Jin X, Zhang D, Zhu G, Shang X, Zhang D, Guo W (2022). Thiamine functions as a key activator for modulating plant health and broad-spectrum tolerance in cotton. The Plant Journal.

[ref-25] Lu J, Breitwieser FP, Thielen P, Salzberg SL (2017). Bracken: estimating species abundance in metagenomics data. PeerJ Computer Science.

[ref-26] Mozafar A (1994). Enrichment of some B-vitamins in plants with application of organic fertilizers. Plant and Soil.

[ref-27] Mozafar A, Oertli JJ (1992). Uptake and transport of thiamin (Vitamin B1) by barley and soybean. Journal of Plant Physiology.

[ref-28] Mozafar A, Oertli JJ (1993). Thiamin vitamin B1: translocation and metabolism by soybean seedling. Journal of Plant Physiology.

[ref-29] Nicot N, Hausman JF, Hoffmann L, Evers D (2005). Housekeeping gene selection for real-time RT-PCR normalization in potato during biotic and abiotic stress. Journal of Experimental Botany.

[ref-30] Olanrewaju OS, Babalola OO (2019). *Streptomyces*: implications and interactions in plant growth promotion. Applied Microbiology and Biotechnology.

[ref-31] Palmer LD, Downs DM (2013). The thiamine biosynthetic enzyme thic catalyzes multiple turnovers and is inhibited by S-adenosylmethionine (AdoMet) metabolites*. Journal of Biological Chemistry.

[ref-32] Sayed SA, Gadallah MAA (2002). Effects of shoot and root application of thiamin on salt-stressed sunflower plants. Plant Growth Regulation.

[ref-33] Schmidhalter U, Kahr G, Evequoz M, Studer C, Oertli JJ (1994). Adsorption of thiamin (vitamin B1) on soils and clays. Soil Science Society of America Journal.

[ref-34] Schmittgen TD, Livak KJ (2008). Analyzing real-time PCR data by the comparative Ct method. Nature Protocols.

[ref-35] Sivadjian J (1953). Synthese et action biologique des vitamines ches les plantes superieures. Bulletin de la Societe Botanique de France.

[ref-36] Srinath E, Pillai S (1966). Observations on some minerals and B vitamins in sewage and sludges. Current Science.

[ref-37] Strzelczyk E, Rozycki H (1985). Production of B-group vitamins by bacteria isolated from soil, rhizosphere, and mycorrhizosphere of pine (*Pinus sylvestris* L.). Zentralblatt fur Mikrobiologie.

[ref-38] Tunc-Ozdemir M, Miller G, Song L, Kim J, Sodek A, Koussevitzky S, Misra AN, Mittler R, Shintani D (2009). Thiamin confers enhanced tolerance to oxidative stress in Arabidopsis. Plant Physiology.

[ref-39] Van Den Heuvel RN, Van Der Biezen E, Jetten MSM, Hefting MM, Kartal B (2010). Denitrification at pH 4 by a soil-derived Rhodanobacter-dominated community. Environmental Microbiology.

[ref-40] Vill A (2023). https://github.com/acvill/bracken_plot.

[ref-41] Von Kocher V, Corti UA (1952). Beiträge zur Kenntnis des Vitamingehaltes von Belebtschlamm aus Abwasserreinigungsanlagen. Schweizerische Zeitschrift für Hydrologie.

[ref-42] Wachter A (2010). Riboswitch-mediated control of gene expression in eukaryotes. RNA Biology.

[ref-43] Wood DE, Lu J, Langmead B (2019). Improved metagenomic analysis with Kraken 2. Genome Biology.

