# Supplementary figures and images for: Thiamin addition to soil increases potato tuber thiamin content under greenhouse conditions
Source: PeerJ. 2026 Jan 29;14:e20684. doi: 10.7717/peerj.20684 (PMC12861137; doi:10.7717/peerj.20684)

A

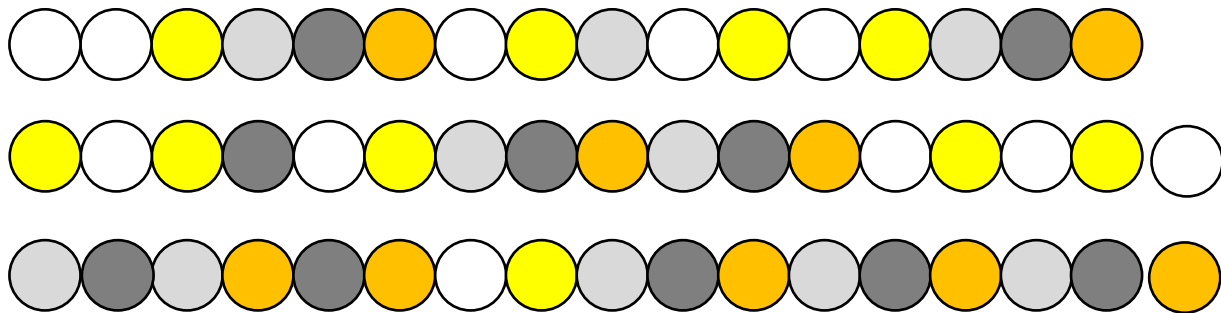

N ←

B

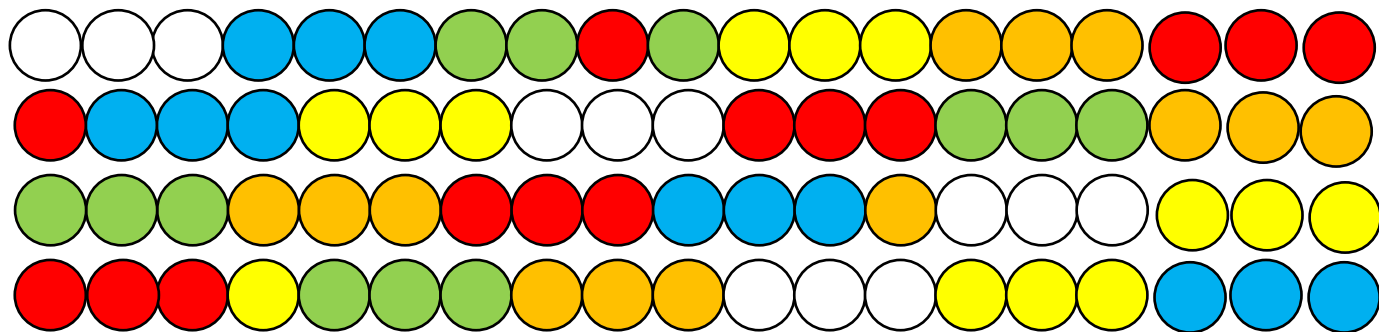

C

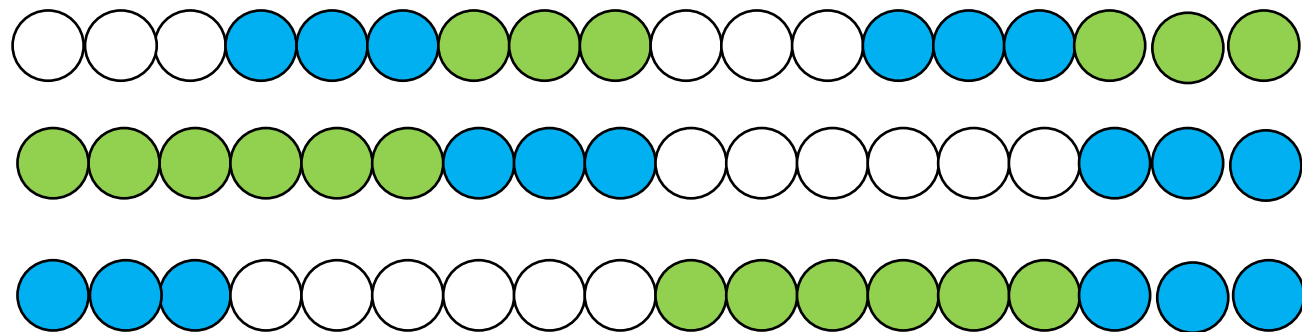

Supplement: Supplemental Information 1 — Each color represents a different thiamin treatment. White, “no pasteurization –no thiamin”; blue, “no pasteurization –20X thiamin”; green, “no pasteurization –50X thiamin”; yellow, “pasteurization –no thiamin”; grey, “pasteurization –1X thiamin”; dark grey, “pasteurization –5X thiamin”; orange, “pasteurization –20X thiamin”; red, “pasteurization –50X thiamin”. [file peerj-14-20684-s001.pdf]

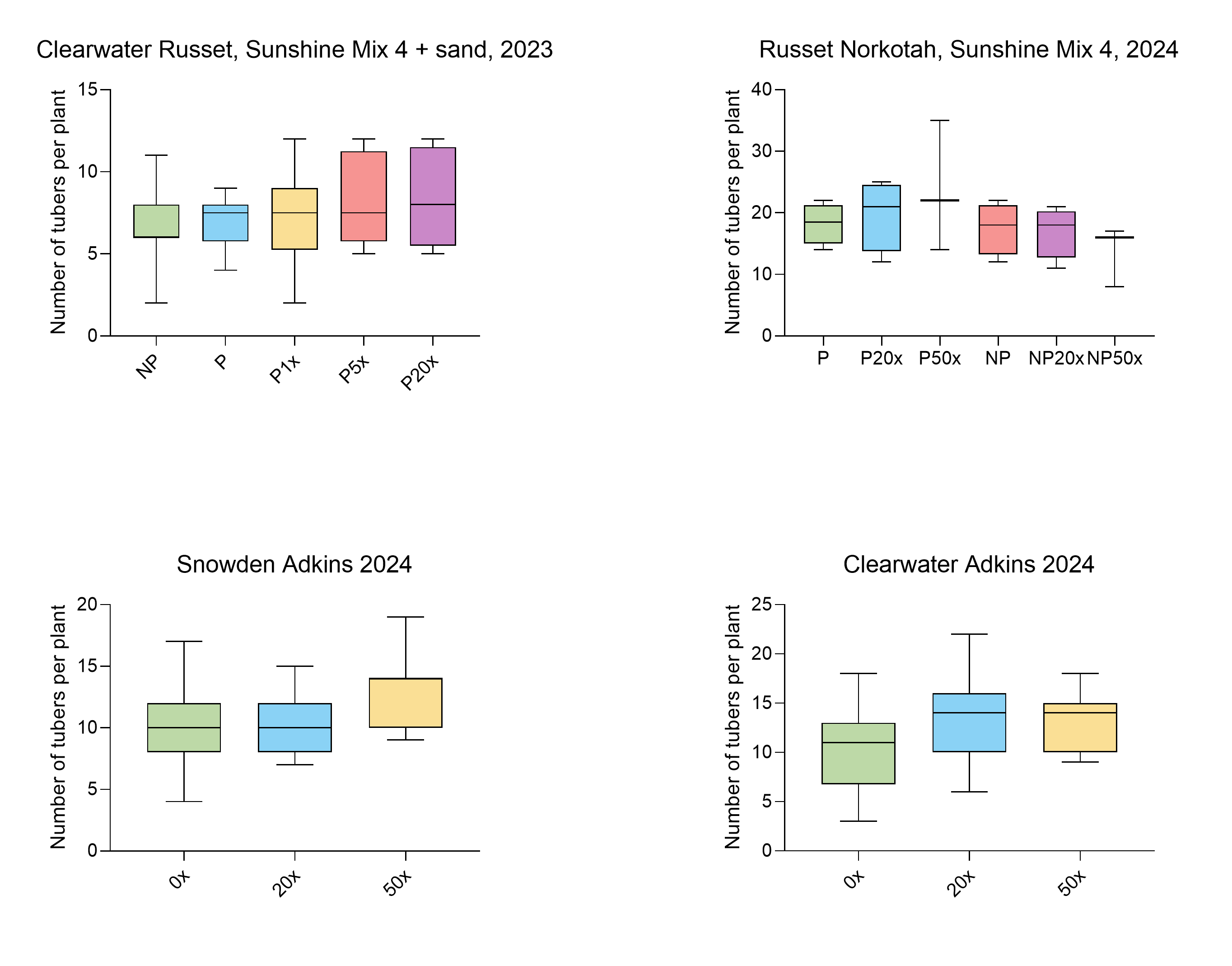

Supplement: Supplemental Information 2 — There was no significant difference in the number of tubers between thiamin treatments. NP, soil not pasteurized. P, pasteurized soil. Graphs were generated in Graphpad Prism version 10.4.1 for Windows (GraphPad Software, Boston, Massachusetts USA ). [file peerj-14-20684-s002.png]

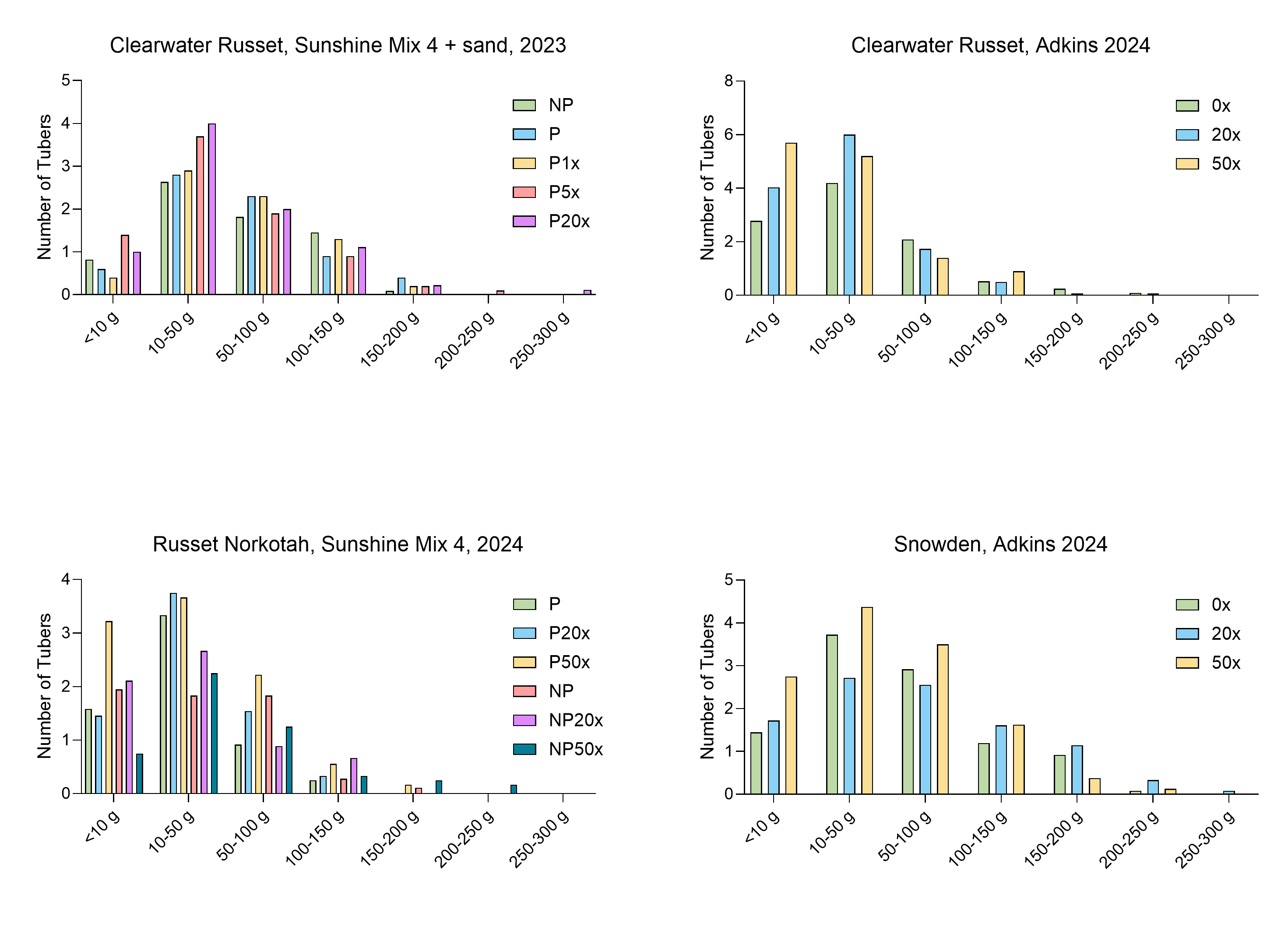

Supplement: Supplemental Information 3 — There was no significant difference in the distribution of tuber weight between thiamin treatments. NP, soil not pasteurized. P, pasteurized soil. [file peerj-14-20684-s003.png]

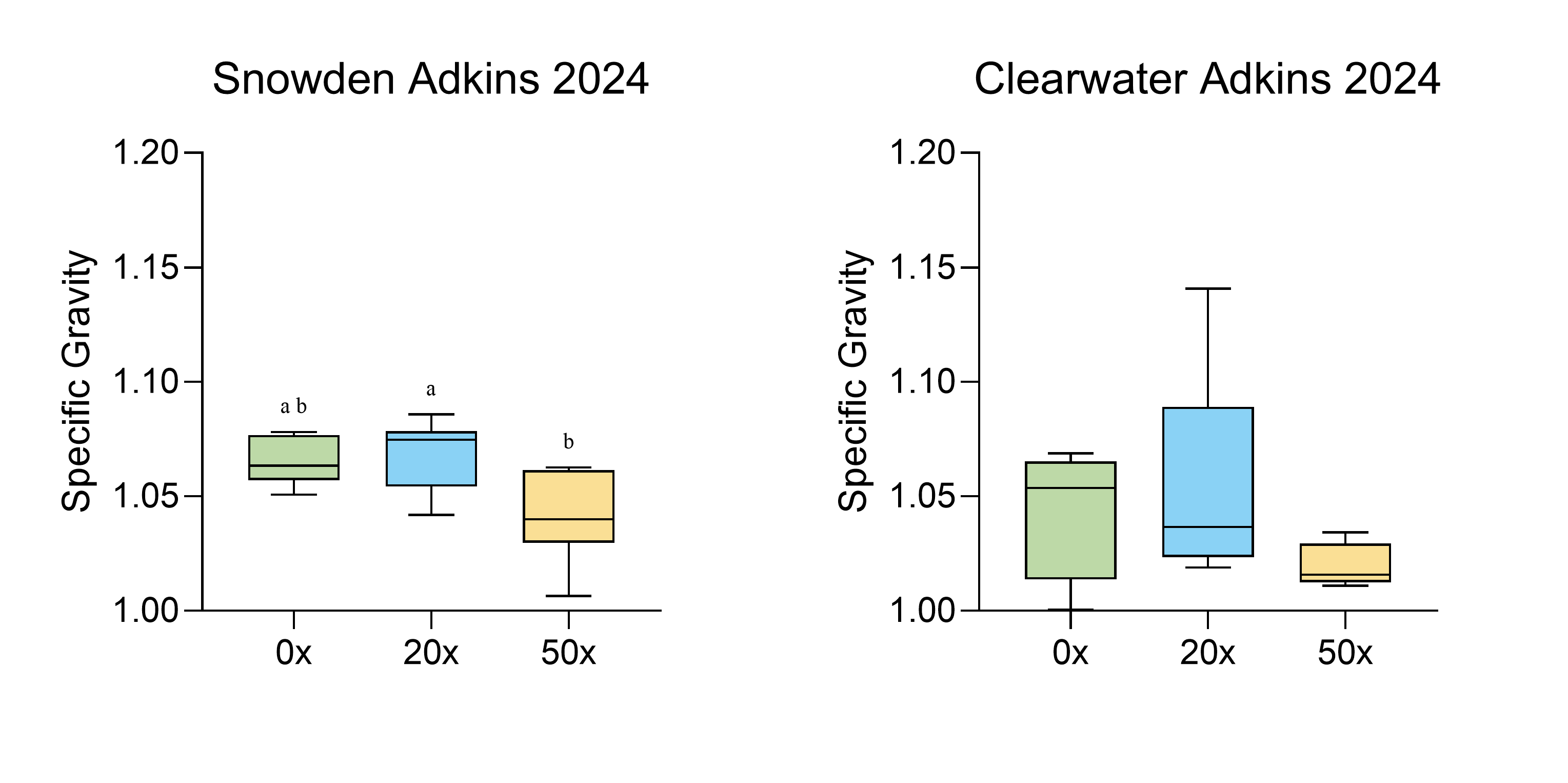

Supplement: Supplemental Information 4 — Identical letters indicate that there were no significant differences between samples as determined by ANOVA (P < 0.05). Graphs were generated in Graphpad Prism version 10.4.1 for Windows (GraphPad Software, Boston, Massachusetts USA). [file peerj-14-20684-s004.png]

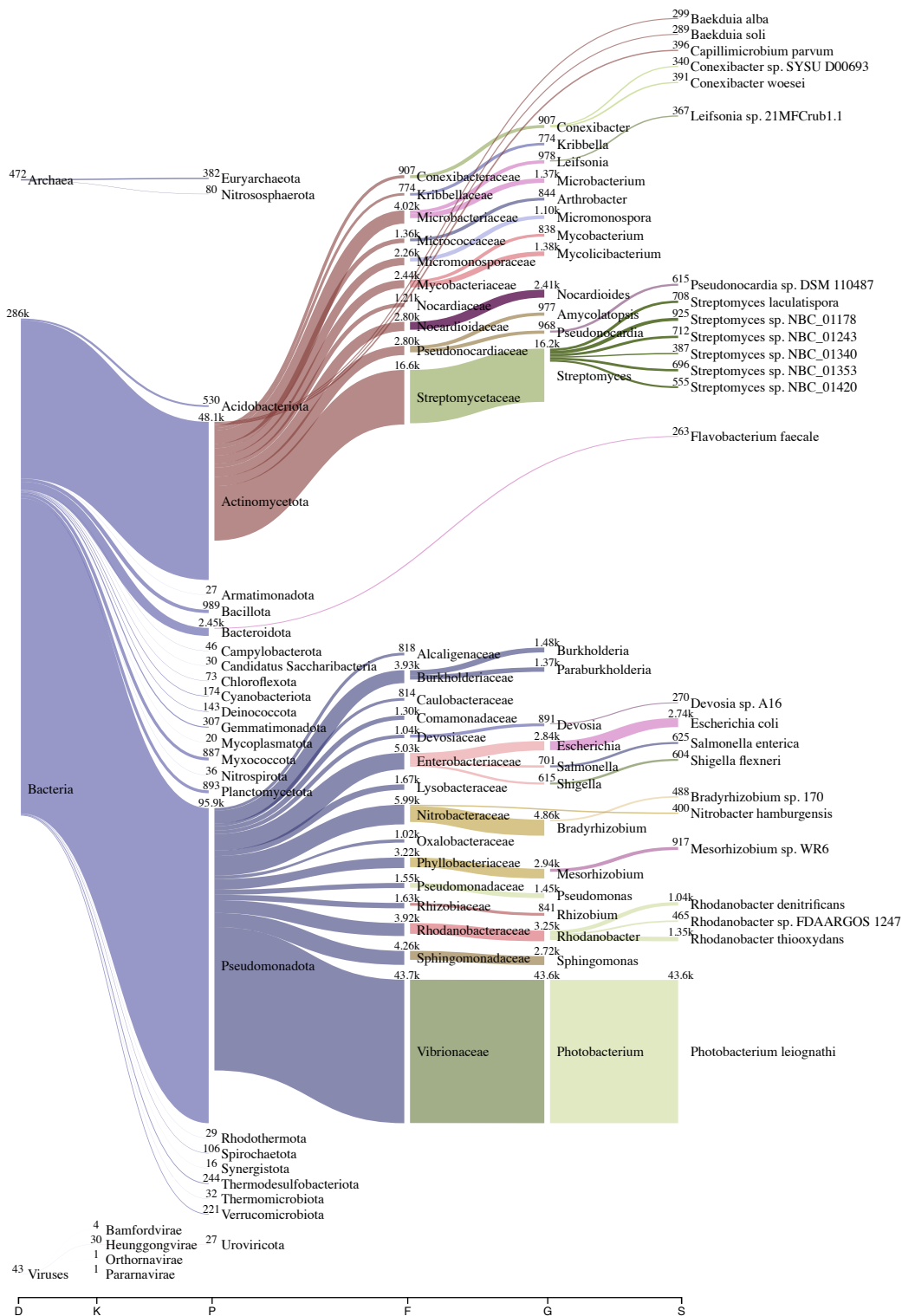

Supplement: Supplemental Information 5 [file peerj-14-20684-s005.pdf]

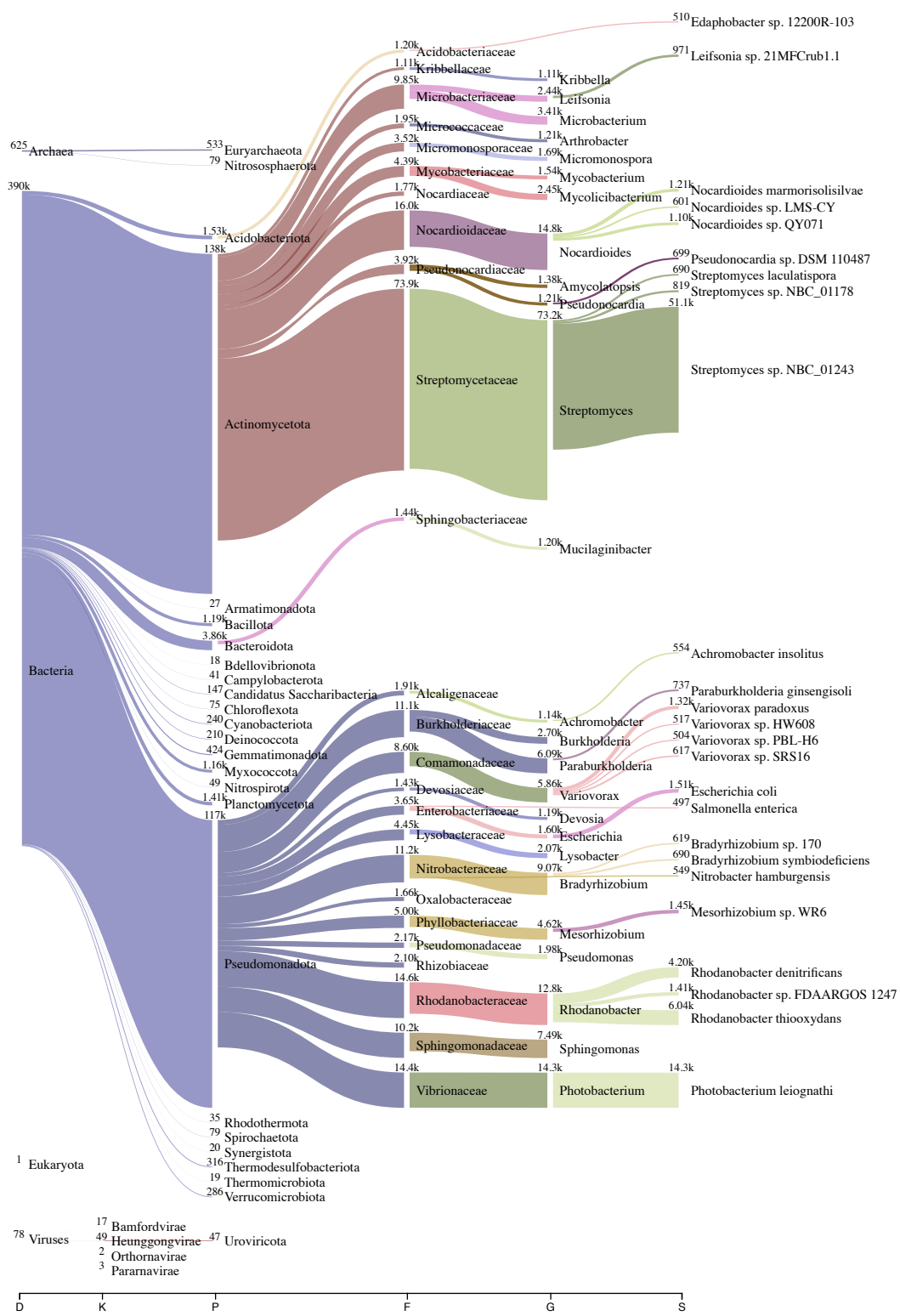

Supplement: Supplemental Information 6 [file peerj-14-20684-s006.pdf]
