# Supplementary material for: Thiamin addition to soil increases potato tuber thiamin content under greenhouse conditions
Source: PeerJ. 2026 Jan 29;14:e20684. doi: 10.7717/peerj.20684 (PMC12861137; doi:10.7717/peerj.20684)
Supplement: Supplemental Information 9 — The sequence of primers, amplicon size, and efficiency are indicated as well as R square of efficiency slope and Cq variation at lower limit (1:200 dilution). [file peerj-14-20684-s009.docx]

**Table S3.** Sequences of primers used for quantitative reverse transcription polymerase chain reaction analysis.

| **Name** | **Sequence (5’ -> 3’)** | **Amplicon size (bp)** | **Efficiency (%)** | **R^2^** | **Cq variation at lower limit (CV%)** |
| --- | --- | --- | --- | --- | --- |
| St-THIC fwd1 | GCGGTGAGATCTACTTGCCA | 135 | 111 | 0.9724 | 1.95 |
| St-THIC rev1 | TCCTACTACCGCCTCCTTGA |  |  |  |  |
| St-IS fwd1 | AAGGAGGCGGTAGTAGGAGC | 89 | 94 | 0.9990 | 2.30 |
| St-IS rev2 | CCCGTTCAGGTTCAAAGGGA |  |  |  |  |
| St-IR fwd2 | AGTGATCACAGCTCCATCGG | 116 | 95 | 0.9614 | 5.74 |
| St-IR rev2 | TGAACAAGGCTGTTGTCTCAGT |  |  |  |  |
| St-THI1 fwd1 | AACCCTGATGTTCAGGTGGC | 159 | 85 | 0.9981 | 1.16 |
| St-THI1 rev1 | CACGTAGTGGTCTTGCTCGT |  |  |  |  |
| 18S fwd | GGGCATTCGTATTTCATAGTCAGAG | 101 | 112 | 0.9641 | 1.24 |
| 18S rev | CGGTTCTTGATTAtTGAAAACATCCT |  |  |  |  |
